# Circulating Tumor Cells in Early Breast Cancer: A 10-Year Follow-Up Update

**DOI:** 10.3390/biomedicines13061278

**Published:** 2025-05-23

**Authors:** Tania Rossi, Emanuela Scarpi, Roberta Maltoni

**Affiliations:** 1 Biosciences Laboratory, IRCCS Istituto Romagnolo per lo Studio dei Tumori (IRST) “Dino Amadori”, 47014 Meldola, Italy; 2 Biostatistics and Clinical Trials Unit, IRCCS Istituto Romagnolo per lo Studio dei Tumori (IRST) “Dino Amadori”, 47014 Meldola, Italy; emanuela.scarpi@irst.emr.it; 3 Outcome Research, Healthcare Administration, IRCCS Istituto Romagnolo per lo Studio dei Tumori (IRST) “Dino Amadori”, 47014 Meldola, Italy; roberta.maltoni@irst.emr.it

**Keywords:** circulating tumor cells, early breast cancer, follow-up, outcome

## Abstract

**Background**: Circulating tumor cells (CTCs) are a rare population of cells considered the seeds of metastasis, detectable in the bloodstream of patients with solid tumors. In breast cancer (BC) their prognostic value is established in the metastatic setting but remains uncertain in early-stage disease. This study provides a 10-year follow-up analysis of disease-free survival (DFS) in a previously described cohort of early-stage BC patients, aiming to evaluate the long-term prognostic significance of CTC detection. **Methods:** As reported in a previous study, 48 patients with stage I–II operable BC were enrolled. CTCs were isolated from peripheral blood using an EpCAM-independent enrichment method followed by DEPArray analysis at baseline (pre-surgery) and six months post-surgery. CTC positivity was defined as the presence of ≥1 CTC. DFS outcomes were assessed over a 10-year follow-up period, and statistical analyses were performed using Kaplan–Meier survival estimates and log-rank tests. **Results:** After 10 years, 3 patients (6.3%) experienced disease relapse, and 2 developed lymphomas. No statistically significant association was observed between CTC presence—either pre-surgery (*p* = 0.13) or post-surgery (*p* = 0.25)—and DFS. Overall, the detection of CTCs using this method did not reliably predict long-term outcomes in this cohort. **Conclusions:** CTC enumeration before and after surgery does not appear to be a reliable prognostic marker for long-term DFS in early-stage BC. Although associated with specific pathological features such as vascular invasion, the role of CTC analysis in this setting remains limited by methodological challenges and cost. Larger, standardized studies are needed to validate the prognostic relevance of conventional and non-conventional CTC populations in early BC.

## 1. Introduction

Circulating tumor cells (CTCs) represent a rare population of cells considered the seeds of metastasis, detectable in the bloodstream of patients with solid tumors. In breast cancer (BC)—one of the most commonly diagnosed cancers in women worldwide and a leading cause of cancer-related mortality—the clinical relevance of CTCs varies depending on the specific setting [1].

In the metastatic context, a pioneering study by Cristofanilli et al. demonstrated that the number of CTCs prior to treatment was an independent predictor of progression-free survival (PFS) and overall survival (OS) in a cohort of 177 patients with metastatic BC. These findings led to FDA approval of the CellSearch enumeration platform for prognostic use in patients with advanced breast, prostate, and colorectal cancers [2]. Subsequent studies have confirmed the prognostic value of CTCs in this setting, establishing a threshold of 5 CTCs/mL to differentiate between metastatic BC cases with favorable and unfavorable outcomes [1]. Moreover, longitudinal monitoring of CTCs during therapy provides more accurate patient stratification in metastatic BC compared to baseline CTC counts alone [3].

In the early-stage setting, however, the landscape is more complex. Enumeration via CellSearch has shown that only 20% of early-stage BC cases have detectable CTCs at diagnosis. In the adjuvant setting, the detection of CTCs before chemotherapy or two years after treatment completion has been independently associated with shorter disease-free survival (DFS) and OS [1]. However, in HER2-positive early BC, some studies have reported an association between CTC count and shorter DFS before neoadjuvant therapy, though not in the adjuvant setting. Consequently, further prospective validation and exploratory studies are required to fully determine the clinical role of CTCs in early-stage BC.

In a previous study published by our group, conducted on a cohort of 48 early-stage BC adjuvant patients (stage I–II), we showed that CTCs were detected in 27.1% of samples before surgery and in 20.1% of samples six months post-surgery using the DEPArray platform. Notably, an association between pre-surgery CTC positivity and vascular invasion was observed [4]. With this communication, we aim to update these findings in a small cohort of adjuvant BC patients after a follow-up of 10 years in terms of DFS.

## 2. Patients After 10-Year Follow-Up

The study involved 48 patients with operable early BC (stages I–II). A detailed summary of the patients’ characteristics, along with the material and methods, are available in our previous publication [4]. Briefly, CTCs were enriched starting from approximately 18–20 mL of peripheral blood collected at baseline (pre-surgery), one month after surgery, and six months after surgery. CTC detection was carried out using the DEPArray platform (Menarini Silicon Biosystems). Samples were considered CTC positive with at least 1 CTC detected.

In this study, we assessed the patients’ statuses after a 10-year follow-up and found that three patients (6.3%) experienced disease relapse during this period. All patient data were collected in May 2024. The clinicopathological features of the patients who had relapsed are detailed in Table 1.

Regarding the clinicopathological characteristics of patients with disease relapse during the 10-year follow-up, two patients (#11 and #39) were hormone receptor (HR)-positive/HER2-negative, while patient #35 had triple-negative breast cancer. Prior to surgery, two of the three patients (#35 and #39) had ≥1 CTCs in peripheral blood. Six months post-surgery, patient #11 converted to CTC-positive, and patient #35 converted to CTC-negative, while CTC analysis was unavailable for patient #39.

**Figure 1:**
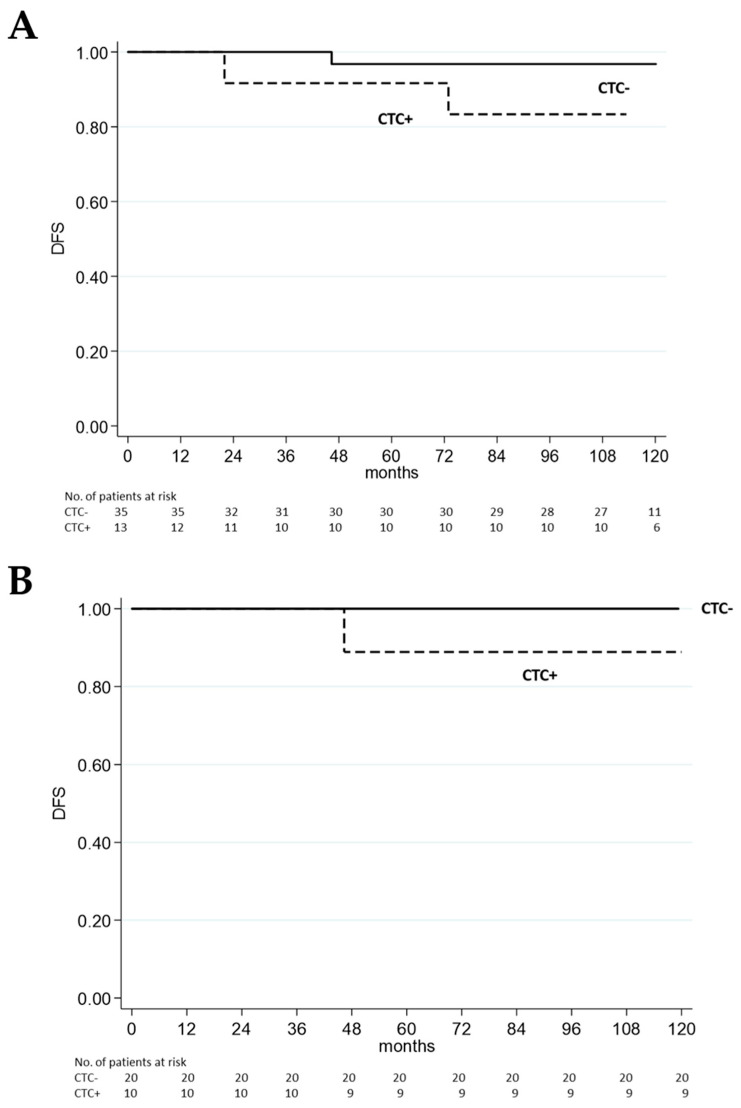
Kaplan–Meier survival curves for DFS. There was no statistical significance in DFS between CTC-positive and CTC-negative patients (**A**) before surgery (*p* = 0.13) and (**B**) six months post-surgery (*p* = 0.25). *p*-values were calculated using the log-rank test applied to Kaplan–Meier survival estimates. For panel B, the analysis was conducted using a six-month landmark approach, with DFS time measured from the landmark to the survival outcome.

Univariate analysis of DFS shows no statistical difference in CTC presence at pre-surgery (Figure 1A) and six months post-surgery (Figure 1B).

Among all the patients in the case series, patient #35 was the only one who died from cancer (51 months after diagnosis and 30 months after disease relapse to the bone).

In addition to tumor relapse, we observed that 2 out of 48 patients were diagnosed with a second primary tumor within the 10-year follow-up. Patient #31 had a diagnosis of HR-positive, HER2-negative early BC subjected to mastectomy and hormonotherapy; she was CTC-negative before surgery, and six months after surgery she presented 1 CTC. Subsequently, 36 months after BC diagnosis, she was diagnosed with a Hodgkin lymphoma (Stage IA) from which she clinically reached complete remission, and at May 2024 she is disease free. Regarding patient #39, 73 months after BC diagnosis she was diagnosed with marginal zone non-Hodgkin B-cell lymphoma (Stage I) localized in the right axilla, concurrent with the diagnosis of BC recurrence in the left supraclavicular node. By May 2024, she finished radiotherapy and achieved complete response.

## 3. Discussion

In recent decades, a growing body of evidence has highlighted the clinical relevance of investigating CTCs as a liquid biopsy approach for early cancer detection, relapse monitoring, therapy response assessment, and therapeutic target identification [5]. In breast cancer, the prognostic value of CTCs is well-established in the metastatic stage, whereas their role in the adjuvant setting remains uncertain. Data from Lucci et al. involving 302 stage I–III breast cancer patients indicated that pre-surgical CTC detection via CellSearch was predictive of early recurrence and reduced overall survival (OS) [6]. Similarly, the SUCCESS study group emphasized the prognostic significance of CTC enumeration both before and after adjuvant therapy in a large prospective trial on early breast cancer patients (pT1-T4, pN0-N3, M0) [7]. Moreover, Sparano et al. demonstrated that the presence of at least one CTC in blood collected five years post-diagnosis provided prognostic information regarding late clinical recurrence [8]. Although CTCs show promise as prognostic biomarkers in non-metastatic breast cancer, further validation studies are necessary to clarify their precise clinical implications. Additionally, given the unique recurrence pattern of breast cancer, which may occur even years or decades later, studies with extended follow-up periods are required.

In a previous study involving a small cohort of 48 stage I–II breast cancer patients, we found that CTC detection using an EpCAM-independent enrichment and DEPArray-based approach was statistically associated with vascular invasion. However, the role of CTCs as predictors of PFS and OS could not be assessed due to incomplete follow-up [4].

In this report, we evaluated CTC detection both before surgery and six months post-surgery, examining its relationship with patient outcomes over a 10-year follow-up period to assess their potential as predictors of late recurrence. In our case series of 48 breast cancer patients in the adjuvant setting, we observed that 3 patients (6.3%) experienced disease relapse within the 10-year follow-up period. Moreover, the detection of CTCs in peripheral blood collected before surgery and six months after surgery did not show a statistically significant correlation and appears unsuitable as a predictor of late recurrence in our case series. This finding is inconsistent with data provided by other studies [6,7], and discrepancies may be attributed to differences in the study setting and methodological approach. More specifically, our case series included patients with breast cancer diagnosed at very early stages (stage I–II), which explains the limited tumor burden in the bloodstream and the low presence of CTCs [9].

This study has several limitations. The major flaw of this study consists of the limited number of patients recruited for this study. Considering that only a small fraction of patients with early-stage BC are CTC positive (20%), and the cut-off is set at 1 CTC per milliliter of blood (whilst a threshold of ≥5 CTCs is used in metastatic disease) [10], enlarged case series are required to obtain robust statistical data for experimental validation study. Another issue regards the method for CTC detection implied in our study: our approach, comprising EpCAM-independent enrichment, cell staining, and DEPArray analysis, is not considered the gold standard approach, which is the CellSearch enumeration system. However, while CellSearch remains the only FDA-approved assay for CTC counting, it fails to identify CTCs with low EpCAM expression and other non-canonical CTC populations, leading to a possible underestimation of CTC. In contrast, our method, as demonstrated by our team and other researchers, successfully identifies CTCs with mesenchymal features and low or absent EpCAM levels, which may be overlooked by the gold standard method [11,12].

Collectively, our findings suggest that the enumeration of CTCs—before and six months after surgery—in stage I–II breast cancer patients should not be used for long term outcome prediction in terms of OS and PFS. Instead, it remains associated with negative tumor-specific prognostic factors, such as vascular invasion.

The enumeration of CTCs in clinical settings is generally limited due to the high cost of analysis, and in conditions such as early-stage breast cancer, their role remains the subject of debate. Moreover, in early-stage breast cancer patients—particularly in the adjuvant setting—the role of CTCs is still uncertain and faces challenges for integration into clinical practice. Looking forward, further validation studies involving larger case series are necessary. Although the CellSearch platform is a well-established system for CTC enumeration, the potential existence of unconventional CTC populations that may not be detected by this method should be considered, following a thorough characterization of their role in the disease.

## Figures and Tables

**Table 1:** Clinicopathological data of the primary tumor of patients’ who experienced tumor relapse within 10-year follow-up period.

ID	ER (%)	PgR (%)	HER2	Histology	Tumor Grade	Type of Surgery	Adjuvant Therapy	Recurrence Site	Time to Relapse (Months)	CTC pre Surgery	CTC Post Surgery
#11	100	5	Not amplified	Ductal	2	Quadrantectomy	TC + H	Mediastinal Node	46.3	−	+
#35	0	0	Not amplified	Ductal	3	Quadrantectomy	AC + T	Bone	22.0	+	−
#39	95	75	Not amplified	Ductal	3	Mastectomy	TC + H	Node	72.9	+	NA

CTC: circulating tumor cell; TC: docetaxel plus cyclophosphamide; AC: adriamycin plus cyclophosphamide; T: taxol; H: hormonotherapy; NA: not available due to sample processing issues; ER: estrogen receptor; PgR: progesterone receptor; HER2: Human Epidermal Growth Factor Receptor 2, #: patient ID.

## Data Availability

The data that support the findings of this study are available from the corresponding author upon reasonable request.

## Abbreviations

The following abbreviations are used in this manuscript: BC: Breast Cancer; CTC: Circulating Tumor Cell; DFS: Disease-Free Survival; OS: Overall Survival; PFS: Progression-Free Survival; FDA: Food and Drug Administration; HR: Hormone Receptor; HER2: Human Epidermal Growth Factor Receptor 2; EpCAM: Epithelial Cell Adhesion Molecule; TC: Docetaxel plus Cyclophosphamide; AC: Adriamycin plus Cyclophosphamide; T: Taxol; H: Hormonotherapy; NA: Not Available.

## References

[1] Reduzzi C., Nicolo’ E., Singhal S., Venetis K., Ortega-Franco A., de Miguel-Perez D., Dipasquale A., Gouda M.A., Saldanha E.F., Kasi P.M. (2024). Unveiling the Impact of Circulating Tumor Cells: Two Decades of Discovery and Clinical Advancements in Solid Tumors. Crit. Rev. Oncol./Hematol..

[2] Cristofanilli M., Budd G.T., Ellis M.J., Stopeck A., Matera J., Miller M.C., Reuben J.M., Doyle G.V., Allard W.J., Terstappen L.W.M.M. (2004). Circulating Tumor Cells, Disease Progression, and Survival in Metastatic Breast Cancer. N. Engl. J. Med..

[3] Magbanua M.J.M., Hendrix L.H., Hyslop T., Barry W.T., Winer E.P., Hudis C., Toppmeyer D., Carey L.A., Partridge A.H., Pierga J. (2021). Serial Analysis of Circulating Tumor Cells in Metastatic Breast Cancer Receiving First-Line Chemotherapy. JNCI J. Natl. Cancer Inst..

[4] Maltoni R., Fici P., Amadori D., Gallerani G., Cocchi C., Zoli M., Rocca A., Cecconetto L., Folli S., Scarpi E. (2015). Circulating Tumor Cells in Early Breast Cancer: A Connection with Vascular Invasion. Cancer Lett..

[5] Alix-Panabières C., Pantel K. (2024). Advances in Liquid Biopsy: From Exploration to Practical Application. Cancer Cell.

[6] Lucci A., Hall C.S., Lodhi A.K., Bhattacharyya A., Anderson A.E., Xiao L., Bedrosian I., Kuerer H.M., Krishnamurthy S. (2012). Circulating Tumour Cells in Non-Metastatic Breast Cancer: A Prospective Study. Lancet Oncol..

[7] Rack B., Schindlbeck C., Fehm T., Schneeweiss A., Lichtenegger W., Beckmann M.W., Friese K., Pantel K., Janni W., Jückstock J. (2014). Circulating Tumor Cells Predict Survival in Early Average-to-High Risk Breast Cancer Patients. JNCI J. Natl. Cancer Inst..

[8] Sparano J., O’Neill A., Alpaugh K., Wolff A.C., Northfelt D.W., Dang C.T., Sledge G.W., Miller K.D. (2018). Association of Circulating Tumor Cells with Late Recurrence of Estrogen Receptor–Positive Breast Cancer. JAMA Oncol..

[9] Lawrence R., Watters M., Davies C.R., Pantel K., Lu Y. (2023). Circulating Tumour Cells for Early Detection of Clinically Relevant Cancer. Nat. Rev. Clin. Oncol..

[10] Thomas-Bonafos T., Pierga J.Y., Bidard F., Cabel L., Kiavue N. (2024). Circulating Tumor Cells in Breast Cancer: Clinical Validity and Utility. Npj Breast Cancer.

[11] Nicolazzo C., Gradilone A., Loreni F., Raimondi C., Gazzaniga P. (2019). Circulating Tumor Cells: Gold in the Waste. Dis. Markers.

[12] Gallerani G., Rossi T., Ferracin M., Bonafè M. (2023). Settling the Uncertainty about Unconventional Circulating Tumor Cells: Epithelial-to-Mesenchymal Transition, Cell Fusion and Trogocytosis. Int. Rev. Cell Mol. Biol..

